# Rare Benign Large Lymphoid Colonic Polyp

**DOI:** 10.1155/2018/5758689

**Published:** 2018-10-28

**Authors:** Yousef Alshamali, Ahmad Al Taleb, Talal Al-Taweel

**Affiliations:** ^1^Department of Medicine, Division of Gastroenterology, University of British Columbia, Vancouver, BC, Canada; ^2^Mubarak Al-Kabeer Hospital, Histopathology Unit, Jabriya, Kuwait; ^3^Haya Al-Habeeb Gastroenterology Center, Mubarak Al-Kabeer Hospital, Jabriya, Kuwait

## Abstract

Benign lymphoid polyps are uncommon lesions of the small bowel and the colon to a lesser degree that are mostly found in children. There are only few reported cases in adults in which the lesions were predominantly polypoid and described as lymphonodular hyperplasia. We present a case of a large benign lymphoid polyp in the transverse colon of a 64-year-old lady who was referred to our care for a history of alteration in her bowel habit and anemia. Colonoscopy showed a 3 cm (Paris 1p) friable polyp which was excised and retrieved. Histopathology examination confirmed its benign nature supported by immunohistochemical studies. Benign lymphoid polyp is a rare condition posing a diagnostic challenge as it can be misinterpreted as a malignant lesion.

## 1. Introduction

Benign lymphoid hyperplasia in the colon is a rare condition. It can present as a single polyp or multiple polypoid lesions. In order to avoid unnecessary intervention or surgery, it is vital yet challenging to differentiate these lesions from malignant lymphoma. This requires careful histological examination and special staining.

## 2. Case Presentation

A 64-year-old lady was referred to the Gastroenterology Clinic for Colonoscopy. She presented with a history of altered bowel habit and iron deficiency anemia. There was no history of abdominal pain, bleeding, or constitutional symptoms. Her past medical and surgical history was unremarkable except for newly diagnosed diabetes. There was no family history of inflammatory bowel disease or gastrointestinal malignancy. Examination revealed an overweight patient but was otherwise noncontributory. Blood tests confirmed iron deficiency anemia.

**Figure 1:**
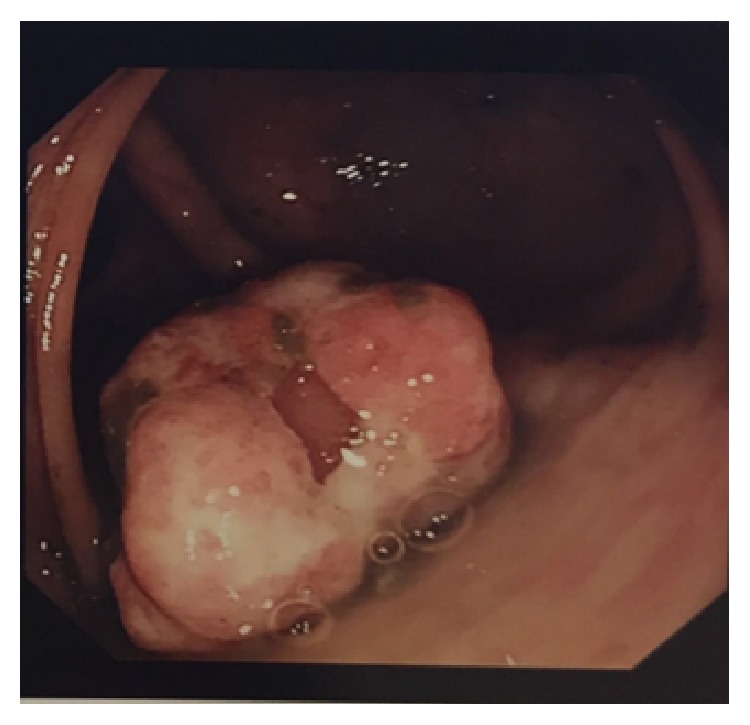
Large friable pedunculated polyp.

**Figure 2:**
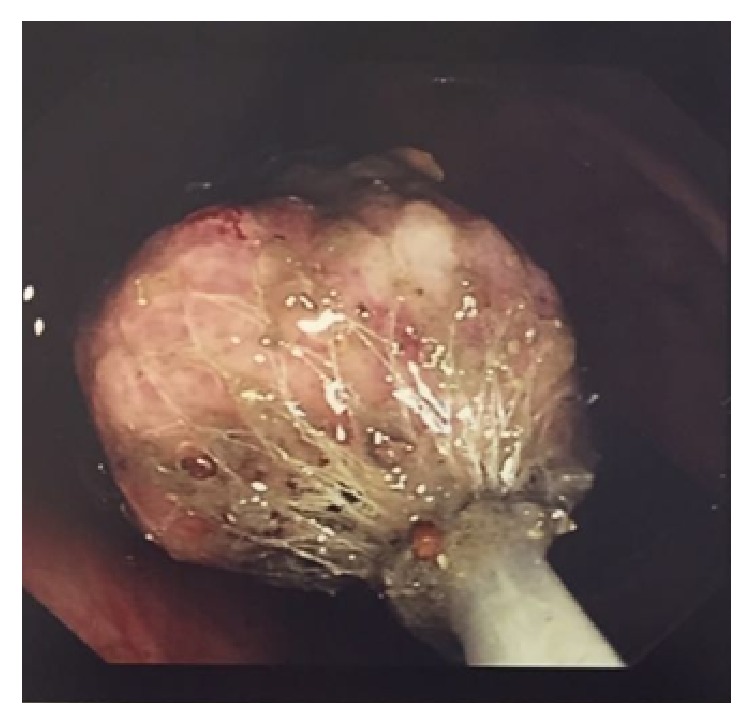
Polyp retrieval.

Colonoscopy showed a large friable pedunculated polyp (Paris 1p) approximately 3 cm in size in the transverse colon (Figure 1). The polyp was excised en bloc in its entirety with snare cautery and retrieved with a retrieval net (Figure 2). 

**Figure 3:**
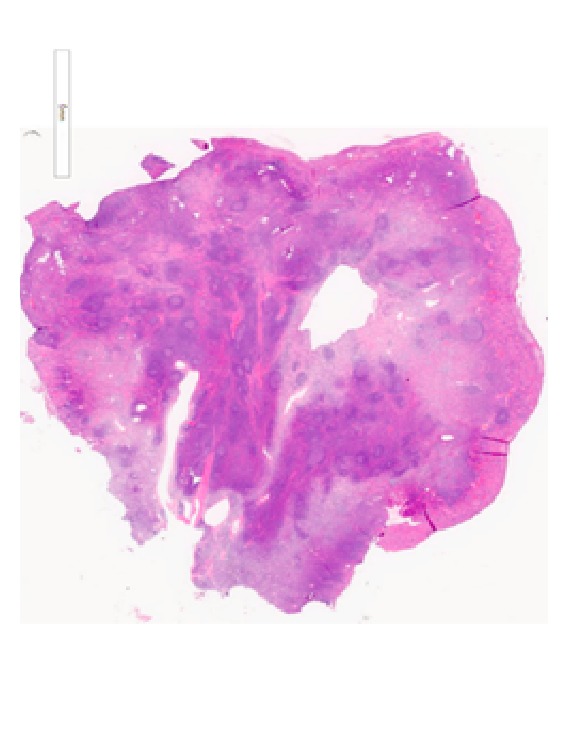
Multiple enlarged lymphoid follicles and prominent germinal centers.

**Figure 4:**
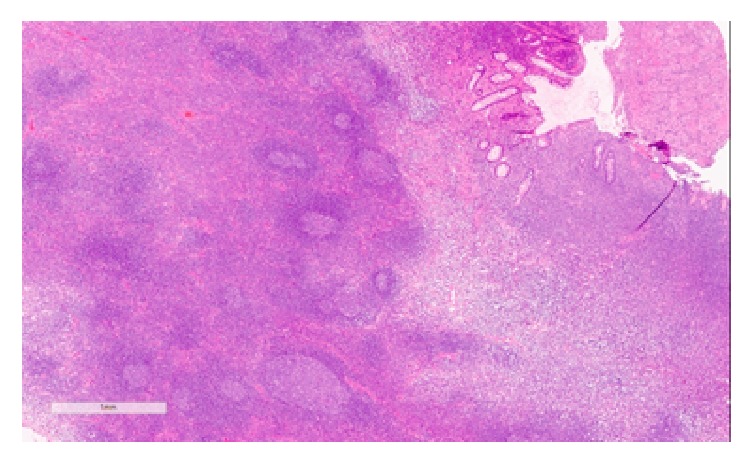
Lymphoid follicles were well-spaced and variably sized and shaped.

Histopathology examination revealed a polyp partially covered by colonic mucosa with areas of erosions and granulation tissue formation. The body of the polyp was composed of hyperplastic lymphoid tissue with multiple enlarged lymphoid follicles and prominent germinal centers. These lymphoid follicles were well-spaced and variably sized and shaped (Figures 3 and 4), and their germinal centers contained typical heterogeneous lymphoid population including tingible body macrophages. CD20 and CD3 immunostains reveal the typical distribution of B-lymphocytes in the follicles and T-lymphocytes in the intervening zones among the follicles, respectively. The overall appearance is reminiscent of nodal follicular hyperplasia, favoring a benign etiology for the polyp; this was confirmed by immunohistochemistry.

## 3. Discussion

Colonic lymphoid hyperplasia is a rare condition especially in adults. It can be localized or diffuse nodular hyperplasia or to a lesser extent a solitary polyp [1]. Polyps are usually sessile and found in the rectum, although they were sometimes reported in the cecum and descending colon [2–4]. A case report by Hong* et al.* documented eighteen cases of rectal lymphoid hyperplasia, eleven of which were polypoidal lesions, three were sessile polyps, and two were nodular lesions [2]. While some patients are asymptomatic, others may present with a variety of symptoms such as rectal bleeding, vague abdominal pain, and altered bowel habit [4–6]. In Hong's review, nine out of the eighteen cases were diagnosed during screening colonoscopy, and seven had rectal bleeding and/or hematochezia. Another case report described a young man who had appendectomy done for one-year history of recurrent right lower quadrant pain with no improvement in his symptoms postoperatively. He was subsequently diagnosed with a large benign lymphoid cecal mass that was invaginating into the ileocecal valve causing pain; this necessitated surgical resection [7]. Furthermore, Taher* et al.* presented a case of a 52-year-old lady who had a large cecal polypoidal mass, measuring 8 cm in size, with severe lymphocytic infiltration and lymphoid follicles. Patient underwent a right hemicolectomy due to potential malignancy; however, histopathological analysis postoperatively confirmed the diagnosis of benign lymphoid hyperplasia [4].

There is an association between lymphoid hyperplasia and immunodeficiency conditions such as common variable immunodeficiency disease, human immunodeficiency virus, and selective IgA deficiency. Also, Giardia and* Helicobacter pylori *infections were reported to be linked to lymphoid hyperplasia in the gastrointestinal tract [8].

**Figure 5:**
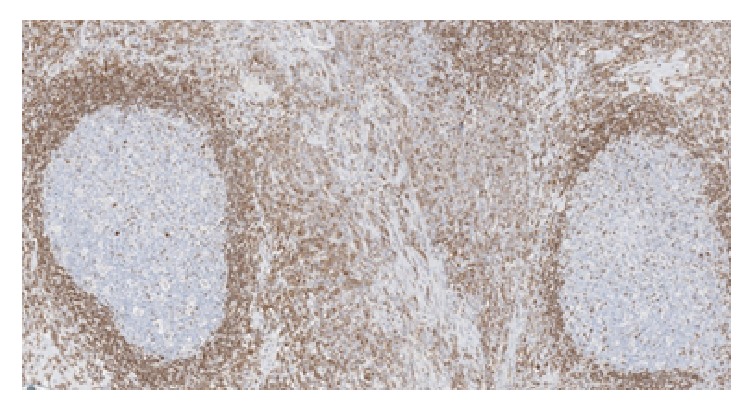
Lymphoid follicles lack Bcl-2.

**Figure 6:**
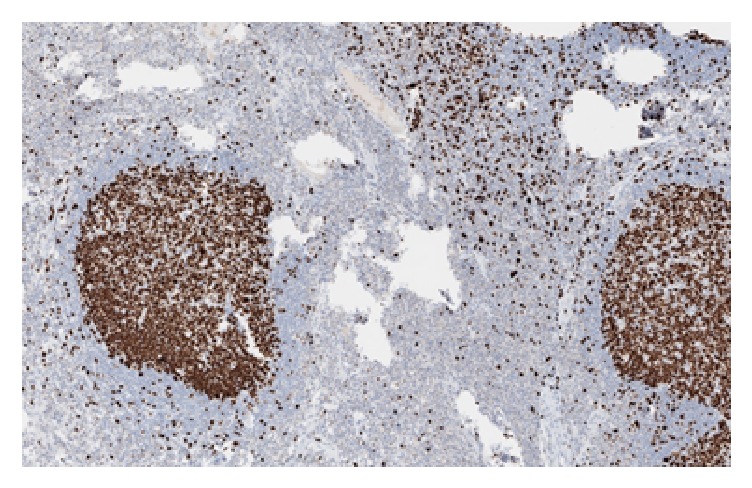
Lymphoid follicles exhibit high ki-67.

Differentiating between benign and malignant polyps is challenging and sometimes requires using ancillary studies such as immunohistochemical analysis and monoclonal immunoglobin gene rearrangement [9]. In the present case, for example, the polyp was covered by normal colonic mucosa and its body was composed of well-spaced lymphoid follicles that varied in size and shape which is suggestive of reactive lymph node. Despite the benign appearance of the polyp, immunohistochemical examination was essential to exclude lymphoid malignancy and avoid misdiagnosis and unnecessary radical treatment. CD20 and CD3 immunostains reveal the typical distribution of B-lymphocytes in the follicles and T-lymphocytes in the intervening zones between the follicles, respectively. Germinal centers of the lymphoid follicles lacked Bcl-2 (Figure 5), while it exhibited high ki-67 proliferating index (Figure 6) ruling out follicular lymphoma. Also, there was no lymphoepithelial lesion seen. The overall histological and immunohistochemical finding are fully consistent with reactive etiology. Therefore, further management other than follow-up colonoscopy as per guidelines was not necessary.

## 4. Conclusion

Colonic lymphoid polyps are rare in adults and can be misdiagnosed as malignant lymphoma. Presentation is variable; patients can be asymptomatic or present with complications requiring surgical intervention. Histological examination with immunohistochemical analysis is essential to differentiate lymphoid hyperplasia from lymphoid malignancy.
